# Lanthanide-based luminescent metal–organic framework as an optical sensing platform for rapid and reliable assessment of antioxidant activity in food samples

**DOI:** 10.1007/s00604-026-08244-8

**Published:** 2026-07-21

**Authors:** Neus Crespí-Sánchez, Francesc Amaro Simeon-Antich, Ernesto Francisco Simó-Alfonso, Enrique Javier Carrasco-Correa

**Affiliations:** 1 Department of Chemistry, University of the Balearic Islands, Cra. de Valldemossa, Km 7.5, 07122 Palma, Spain; 2 CLECEM Group, Department of Analytical Chemistry, Faculty of Chemistry, University of València, Avenida Vicent Andrés Estellés, 19, 46100 Burjassot, València Spain

**Keywords:** Luminescent metal–organic frameworks, Fluorescence detection, Total antioxidant capacity, Food analysis screening, Green Analytical Chemistry

## Abstract

A rapid and sensitive fluorescence-based method for antioxidant capacity assessment was developed using a lanthanide-based metal–organic framework (LVMOF-1). The Eu^3+^-MOF, incorporating an electron-deficient viologen ligand, enables host–guest interactions with phenolic antioxidants, leading to a quantitative quenching of a composite optical signal driven by a resonance convolution of native Eu^3+^ photoluminescence and second-order scattering, inner filter effect and colloidal modulation. Using gallic acid as a model compound, the system exhibited a non-linear Langmuir-type response that was successfully linearized through logarithmic transformation, enabling reliable quantification of total antioxidant capacity over an extended concentration range. The method showed excellent analytical performance, with a limit of detection of 0.0077 mg L^–1^, good linearity (R^2^ = 0.992), and reproducibility below 7% RSD. Compared to conventional assays (Folin–Ciocalteu, DPPH, ABTS, and FRAP), it provided higher sensitivity, shorter analysis time (≤ 2 min), and improved robustness in complex matrices, inherently minimizing color-induced spectral interferences due to the high sensitivity of fluorescence detection. Application to honey, tea, and wine samples showed very strong correlations with established methods (Pearson r = 0.947–0.996). These results were further supported by Bland–Altman analysis, which confirmed good overall agreement with method-dependent differences, and principal component analysis (PCA), which revealed a common underlying antioxidant capacity dimension. Additionally, the LVMOF-1 assay showed the best greenness (AGREE score 0.59) and lowest cost (0.63 € per sample), highlighting its potential as a sustainable alternative for routine antioxidant screening.

**Graphical Abstract:**

**Figure:**
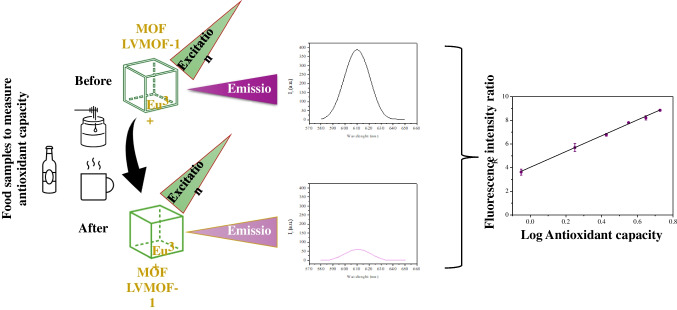

**Supplementary Information:**

The online version contains supplementary material available at 10.1007/s00604-026-08244-8.

## Introduction

Oxidative stress originates from an imbalance between the generation of reactive species, which is involved in the onset and progression of multiple chronic and degenerative disorders. To counteract oxidative damage, living organisms rely on a complex antioxidant network capable of neutralizing radicals by electron or hydrogen donation and, in some cases, by chelating redox-active metals to inhibit chain reactions. While dietary antioxidants are widely recognized for counteracting oxidative stress and preventing cellular damage in biological systems [1, 2], their evaluation in the food and nutraceutical field is of particular interest because these compounds contribute significantly to product stability and overall quality. Accordingly, there is a growing trend to source antioxidants from natural materials, including edible plants and agro-industrial residues, aligning with sustainability goals and circular-economy strategies [3–6].

Antioxidants comprise a broad and varied group of compounds that differ in their mechanisms of action, whether enzymatic or non-enzymatic, as well as in their physicochemical characteristics, such as solubility in aqueous or lipid environments, and their structural diversity, including vitamins, polyphenols, carotenoids, and other phytochemicals. In any case, beyond identifying individual antioxidant compounds, assessing antioxidant activity is a key step for screening raw materials, extracts, and finished products. Although chromatographic and spectroscopic techniques (e.g., HPLC and UV–Vis/fluorescence spectroscopy) enable accurate identification and quantification of specific molecules, routine evaluation is more commonly carried out using antioxidant capacity assays because they are fast, simple, and inexpensive [7–9]. These assays are typically classified according to their dominant mechanism as electron-transfer (ET) methods, such as Folin–Ciocalteu and FRAP, or radical-based assays such as DPPH and ABTS, which may involve both ET and hydrogen-atom-transfer (HAT) mechanisms [7, 10–13] Among these methods, ET-based assays are the most widely used for estimating antioxidant capacity. However, despite their popularity, these conventional approaches present several limitations, including sensitivity to pH and solvent composition, relatively long reaction times, and susceptibility to matrix interferences caused by pigments, sugars, turbidity, or metal ions in food samples [14]. In addition, because these assays rely on different chemical principles, the antioxidant capacity values obtained are often not directly comparable. Consequently, there is an urgent analytical need to focus on developing alternative, fast, and robust analytical approaches that provide shorter response times, improved robustness against matrix effects, and simplified analytical procedures while maintaining reliable analytical performance in complex matrices.

In recent years, advanced functional materials have emerged as promising platforms for chemical sensing in analytical chemistry. Among them, metal–organic frameworks (MOFs) are porous hybrid materials composed of metal ions or metal clusters interconnected by organic ligands to form highly ordered crystalline networks. Owing to their exceptionally high surface area, tuneable pore size, and versatile chemical functionality, MOFs have attracted considerable attention for applications in catalysis, adsorption, separation, and sensing [15–17]. One particularly attractive feature of MOFs for sensing applications is their ability to host guest molecules within their porous structure and generate measurable physicochemical responses upon interaction. In many cases, analyte molecules can diffuse into the pores and interact with the framework through hydrogen bonding, π interactions, or charge-transfer processes [18, 19]. These host–guest interactions can lead to measurable optical responses, particularly through the modulation of their intrinsic photoluminescent or light-scattering, inner effect or colloidal modulation properties [20]. For this reason, luminescent and colloidally stable MOFs have emerged as versatile sensing materials for the detection of a wide variety of analytes, including metal ions, small organic molecules, and bioactive compounds.

Among fluorescent MOFs, lanthanide-based MOFs (Ln-MOFs) are particularly attractive because lanthanide ions display characteristic luminescence properties such as narrow emission bands, long emission lifetimes, and large Stokes shifts [21, 22]. These properties provide high signal resolution and reduce spectral interferences, which are advantageous for analytical sensing applications [23]. In Ln-MOF systems, luminescence typically arises from ligand-to-metal energy transfer processes, often referred to as the antenna effect, in which the organic ligand absorbs the excitation energy and transfers it to the lanthanide center, generating the characteristic emission of the metal ion. In colloidal suspensions, however, these native electronic transitions may convolute with instrumental light-scattering phenomena, giving rise to complex composite optical profiles.

Importantly, the luminescence of these materials can be modulated by the presence of guest molecules capable of interacting with either the ligand or the metal centre. Such interactions may induce fluorescence quenching or enhancement depending on the nature of the analyte and the interaction mechanism [24]. In the case of antioxidant compounds, many molecules of interest, particularly phenolic antioxidants, are electron-rich aromatic systems containing hydroxyl groups capable of participating in electron-donation processes, hydrogen bonding, or π–π interactions [2]. These properties make them suitable candidates for interacting with electron-deficient sensing frameworks, potentially modulating their overall optical response. In this context, MOFs incorporating electron-deficient ligands represent promising platforms for the detection of phenolic antioxidants. Viologen-based ligands, in particular, are well known for their strong electron-accepting character and their ability to participate in charge-transfer interactions with electron-rich aromatic compounds [23]. When incorporated into MOF structures, these ligands can promote strong electronic interactions with phenolic molecules, potentially modifying the photophysical and colloidal properties of the framework.

Despite these promising characteristics, the application of MOF-based optical platforms for evaluating antioxidant capacity remains relatively underexplored. Only a limited number of studies have investigated the use of MOF-based luminescent systems for monitoring interactions between antioxidant molecules and porous frameworks [25], highlighting the need for further research in this area. Consequently, a practical gap remains in routine quality control: conventional assays still require lengthy incubation times, generate significant chemical waste, and often suffer from high measurement variability when applied to complex samples.

In this work, the synthesis and characterization of a europium-based metal–organic framework, LVMOF-1, designed for spectroscopic detection of antioxidant activity is reported. LVMOF-1 features microporous channels and incorporates a viologen-type ligand (N,N′-bis(3,5-dicarboxybenzyl)−4,4′-bipyridinium dichloride, [H_4_L]Cl_2_), which is electron-deficient and therefore capable of interacting strongly with electron-rich aromatic antioxidants. Gallic acid was selected as a representative model analyte and a proxy for the broader class of phenolic antioxidants due to its universal benchmark status in conventional colorimetric testing [26, 27], thereby ensuring a direct performance comparison with established methodologies. Using this compound, the composite optical response of LVMOF-1 was investigated and applied to the determination of total antioxidant capacity. The analytical performance of the proposed method was evaluated and compared with conventional antioxidant assays (Folin–Ciocalteu, DPPH, ABTS, and FRAP). Finally, the applicability of the method was demonstrated through the analysis of different food samples, including honey, tea, and wine, highlighting the potential of LVMOF-1 as a rapid and reliable platform for antioxidant screening in complex matrices. Compared with traditional colorimetric assays and conventional luminescent sensors, the novelty of the proposed analytical method lies in its streamlined, single-vessel operational protocol that eliminates the need for tedious incubation periods or hazardous organic solvents. By exploiting an immediate spectroscopic response driven by the structural and optical features of the framework, this approach seeks to establish a highly sustainable, low-cost, and reproducible testing alternative tailored to the practical demands of routine screening.

## Experimental section

### Reagents and materials

Folin–Ciocalteu reagent, hydrochloric acid 37% (HCl), iron(III) chloride hexahydrate (FeCl_3_·6H₂O), acetonitrile (ACN), methanol (MeOH) potassium persulfate (K₂S₂O₈), and glacial acetic acid were obtained from VWR Chemicals (Radnor, PA, USA). Anhydrous sodium carbonate was purchased from Fisher Chemical (Loughborough, UK), and anhydrous sodium acetate from PROBUS (Barcelona, Spain). 2,4,6-Tris(2-pyridyl)−1,3,5-triazine (TPTZ) and 4,4'-bipyridine were supplied by TCI Chemicals (Tokyo, Japan). 2,2-Diphenyl-1-picrylhydrazyl (DPPH) was obtained from Alfa Aesar (Ward Hill, MA, USA), and 2,2'-azino-bis(3-ethylbenzothiazoline-6-sulfonic acid) diammonium salt (ABTS) from GenoChem World (Shanghai, China). Acetone was purchased from Scharlab (Sentmenat, Spain). Europium(III) chloride hexahydrate (EuCl₃·6H₂O) was obtained from Thermo Scientific (Waltham, MA, USA), dimethyl 5-(bromomethyl)isophthalate from Pharmatech (Mumbai, India), and gallic acid from Across Organics (Geel, Belgium). All aqueous solutions were prepared with deionized water obtained using a Crystal B30 EDI deionizer (Adrona, Riga, Latvia).

### Food samples and sample solutions and extracts

The samples analysed in this study included three honeys, three teas, and three wines. Their characteristics are summarized in Table 1.

**Table 1:** Sample Description

Food	Sample	Description
Honey	H1	Thyme honey (Santiago de la Espada, Jaén, Spain)
	H2	Multifloral honey (Lucena, Castellón, Spain)
	H3	Thyme and rosemary honey (Catí, Castellón, Spain)
Tea	T1	Green tea Gyokuro Asahi (Japan; distributed by East West Company Spain, S.L.)
	T2	Green tea, Camellia sinensis leaves (distributed by Pompadour Ibérica, S.A., Spain)
	T3	Red tea, Camellia sinensis leaves (distributed by Pompadour Ibérica, S.A., Spain)
Wine 1	W1	Red wine, Viñas Viejas de Paniza, Garnacha (Cariñena 2020, Spain)
	W2	White wine, Pata Negra, Sauvignon Blanc Verdejo (Rueda 2022, Spain)
	W3	Sparkling rosé wine, Peñascal (2020, Valladolid, Spain)

Honey samples were prepared by accurately weighing 800 mg of each sample and dissolving it in 10 mL of ultrapure water. The solutions were sonicated in an ultrasonic bath for 5 min and filtered through PTFE syringe filters (0.45 µm pore size, 13 mm diameter). In addition, other solutions were prepared to ensure that the resulting analytical signals values fell within the calibration range in the respective assays. Tea samples were extracted by infusing 200 mg of ground tea in 10 mL of freshly boiled ultrapure water for 10 min. The extracts were cooled to room temperature and filtered through PTFE syringe filters (0.45 µm pore size, 13 mm diameter). All tea extracts were subsequently diluted tenfold with ultrapure water prior to analysis. Wine samples were analysed without prior treatment, except for Red Wine 1 (Viñas Viejas de Paniza, Garnacha), which was diluted 1:10 with ultrapure water before the assays.

### Instrumentation

The crystalline structure of the materials was analysed by powder X-ray diffraction (PXRD) using a D8 Advance diffractometer (Bruker) equipped with a copper anode, a nickel filter, and a Lynxeye one-dimensional energy-dispersive detector. Nitrogen adsorption–desorption isotherms were measured at 77 K using a TriStar II 3020 surface area and porosity analyser (Micromeritics), with helium employed for dead volume calibration. The Brunauer–Emmett–Teller (BET) model was applied to calculate the specific surface area of LVMOF-1. Fourier-transform infrared (FT-IR) spectra were recorded with a Vertex 80v spectrometer (Bruker). FT-IR was used to characterize the functional groups present in both LVMOF-1 and the ligand [H_4_L]Cl_2_, as well as to study the interactions between gallic acid and LVMOF-1. Conventional antioxidant capacity assays were performed by measuring UV–Vis absorbance using a V-650 double-beam spectrophotometer equipped with Spectra Manager II software (Jasco). Fluorescence measurements were performed using a FP-6200 spectrofluorometer (Jasco) to monitor the composite optical response of LVMOF-1 and to evaluate its interaction with gallic acid during calibration experiments.

### Conventional antioxidant capacity assays

Calibration curves were prepared in triplicate for each method using aqueous gallic acid standard solutions (0.5–10 mg L^–1^), made from a stock solution of 200 mg L^–1^ in ultrapure water which was prepared freshly every two days to avoid degradation. All sample measurements were performed in triplicate. The conventional antioxidant capacity assays (Folin–Ciocalteu, DPPH, ABTS, and FRAP) were carried out following previously reported procedures [26–28], with detailed experimental protocols provided in the Supplementary Information.

### Synthesis of the fluorescent LVMOF-1

First, the ligand was synthesized following the procedure described by Gong et al. [28]. Briefly, a solution of 4,4′-bipyridine (1.00 g, 6.40 mmol) and dimethyl 5-(bromomethyl)isophthalate (3.67 g, 12.8 mmol) in 15 mL of ACN was refluxed for 24 h under ambient atmosphere. After cooling, the resulting solid was collected by filtration, washed with ACN, and dried under vacuum to yield the tetramethyl ester intermediate [Me_4_L]Br_2_ as a yellow solid. Subsequently, [Me_4_L]Br_2_ (1.00 g, 1.37 mmol) was dissolved in 30 mL of 37% HCl and refluxed for 48 h. The solution was cooled to room temperature, stored at 2 °C for 12 h, and the resulting N,N′-bis(3,5-dicarboxybenzyl)−4,4′-bipyridinium dichloride ([H₄L]Cl₂) precipitate was collected by filtration, washed with water and acetone, and dried under vacuum. Next, the LVMOF-1 was synthesized using a solvothermal method as reported by Gong et al. [23]. A mixture of EuCl_3_·6H_2_O (0.049 g, 0.19 mmol), [H_4_L]Cl_2_ (0.050 g, 0.085 mmol), 2 mL of ACN, and 1 mL of water was sealed in a vial inside a Teflon-lined autoclave and heated at 120 °C for 3 days. Afterwards, the Teflon-lined autoclave was removed from the oven and allowed to cool down to room temperature. The resulting pale-yellow crystals were recovered by centrifugation at 6000 rpm for 10 min, washed with water, and dried at 60 °C.

### Study of interactions with gallic acid

The interaction between LVMOF-1 and gallic acid was studied by preparing a gallic acid@LVMOF-1 composite. For this, 10 mg of LVMOF-1 were mixed with 1.5 mL of an aqueous gallic acid solution (2000 mg L^–1^) and shake at room temperature for 24 h. The solid was collected by centrifugation, washed with water, air-dried, and analysed by FT-IR.

### Antioxidant sensing methodology using the LVMOF-1

A stock dispersion of LVMOF-1 was prepared at a concentration of 750 mg L^–1^ by adding 150 mg of finely ground LVMOF-1 to 200 mL of ultrapure water. This mixture was sonicated for 2 h, using an ice-water bath to maintain the dispersion at room temperature and prevent thermal degradation, to ensure a homogeneous dispersion. The calibration was performed using the sequential standard addition method. In each quartz cuvette, 3 mL of the LVMOF-1 dispersion were placed, and successive additions of 10 μL of a 200 mg L^–1^ gallic acid aqueous solution were made. After each addition, the solution was left to rest for 2 min under static conditions to allow the analytical signal to stabilize. The composite optical signal was measured at 615 nm (λ_exc_ = 306 nm with excitation and emission slit widths set at 5 nm, and the photomultiplier tube (PMT) voltage configured in medium mode). Since the quantification of antioxidant capacity was performed using an in-cuvette sequential standard addition method, to ensure absolute accuracy in the calibration curve, the concentrations of both the GA and the LVMOF-1 were mathematically calculated for each successive addition step (i) to account for progressive dilution, according to the following equations:

1 $${C}_{GA,i}=\frac{{C}_{stock, GA}\times {V}_{add,i}}{{V}_{0}+{V}_{add,i}}$$

2 $${C}_{MOF,i}=\frac{{C}_{0}\times {V}_{0}}{{V}_{0}+{V}_{add,i}}$$

where V_0_ is the initial volume of the MOF dispersion in the cuvette, C_0_ is its initial concentration of the LVMOF-1, C_stock, GA_ is the concentration of the added GA standard solution, and V_add,i_ represents the cumulative volume of standard solution added at step i.

For the samples, 10 μL of the sample were added to the LVMOF-1 dispersion and the same protocol was followed without the in-cuvette sequential standard protocol. All measurements were performed in triplicate.

## Results and discussion

### Characterization of LVMOF-1

**Fig. 1:**
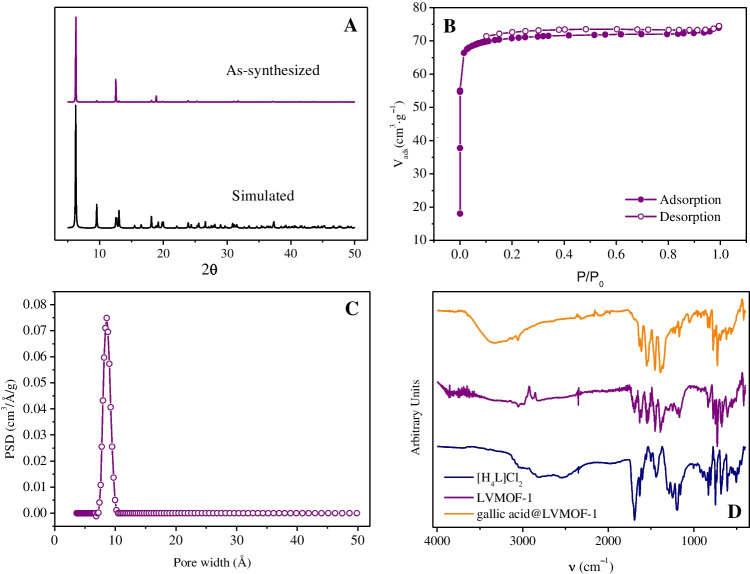
(A) PXRD pattern of the crystalline structure of the synthesized LVMOF-1, as-synthesized and simulated. (B) Nitrogen adsorption–desorption isotherm and (C) pore size distribution of the synthesized LVMOF-1. (D) FTIR spectra of gallic acid@LVMOF-1 (orange), synthesized LVMOF-1 (purple), and the ligand [H_4_L]Cl_2_ (blue)

The synthesized MOF was characterized by PXRD and N_2_ adsorption–desorption (BET) analysis. The PXRD pattern (Fig. 1A) exhibits intense and well-defined diffraction peaks, indicating the high crystallinity of the synthesized material. Crucially, the excellent overlap between the experimental Bragg reflections and the simulated profile confirms the successful formation and phase purity of LVMOF-1, matching the structure previously reported by Gong et al. [23], supporting the proposed interaction of LVMOF-1. Nitrogen adsorption–desorption measurements further confirmed the porous nature of the material. As shown in Fig. 1B, the isotherm corresponds to a classic Type I profile, characterized by a sharp gas uptake at low relative pressures (P/P_0_ < 0.1), which is highly characteristic of strictly microporous architectures with no significant mesoporosity or secondary hysteresis. Underpinning this framework architecture, the material exhibits a specific surface area of 217 m^2^ g^–1^ and a pore volume of 0.111 cm^3^ g^–1^. Consistent with this observation, pore size distribution analysis (Fig. 1C) reveals a single, sharp population of pore diameters in the range of 7–10 Å, in perfect mathematical agreement with the microporous architecture suggested by the adsorption isotherm.

The FTIR spectra (Fig. 1D) further support the successful formation of LVMOF-1 through comparison with the spectrum of the free viologen-based ligand. In the ligand spectrum (blue spectrum), a strong band is observed around 1700 cm^–1^, corresponding to the stretching vibration of the carbonyl (C = O) group of the carboxylic acid moieties. A broad absorption band between 3200 and 3600 cm^–1^ is also observed and can be attributed to O–H stretching vibrations associated with hydrogen-bonded carboxylic groups. In addition, aromatic C = N stretching vibrations appear in the 1550–1650 cm^–1^ region, while bands between 1400 and 1500 cm^–1^ are mainly associated with aromatic C = C stretching and C–H bending modes.

Upon coordination with Eu^3+^ to form the MOF (purple spectrum), significant spectral changes are observed. The disappearance of the strong C = O stretching band around 1700 cm^–1^ and the appearance of new bands in the 1600–1400 cm^–1^ region is highly indicative of indicates the deprotonation of the carboxylic acid groups and their coordination to the europium centres as carboxylate ligands. These bands can be principally assigned to the asymmetric and symmetric stretching vibrations of coordinated carboxylate groups, which likely overlap with the core skeletal vibrations of the aromatic viologen ring. Additionally, the appearance of bands in the low-frequency region below 500 cm^–1^ can be tentatively attributed to Eu–O vibrations, further supporting the formation of the metal–ligand framework.

The interaction of LVMOF-1 with gallic acid was also investigated. Upon addition of gallic acid (2000 mg L^–1^), the material undergoes a rapid colour change from pale yellow to orange coloration, suggesting the occurrence of charge-transfer interactions between the electron-deficient viologen moieties of the framework and the electron-rich phenolic compound [23]. This interaction is further supported by the FTIR spectrum of the gallic acid@LVMOF-1 composite (orange spectrum, Fig. 1D). Free gallic acid exhibits a sharp O–H stretching band in the 3400–3600 cm^–1^ region; however, after interaction with the MOF, this characteristic profile shifts toward lower wavenumbers (3200–3400 cm^–1^), suggesting the formation of hydrogen-bonding interactions. Additionally, slight changes in the intensity and position of the carboxylate and Eu–O bands indicate interactions between gallic acid and the MOF framework, likely involving hydrogen bonding and π–π or charge-transfer interactions with the viologen units.

### Optical and spectroscopic properties of LVMOF-1

**Fig. 2:**
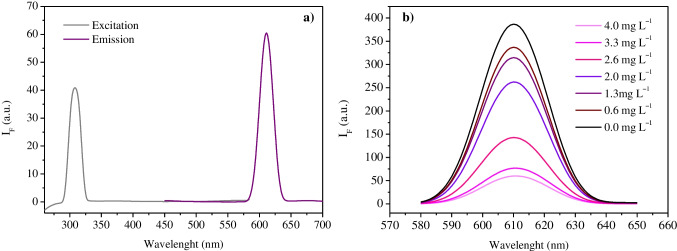
(**A**) Excitation and emission spectra of LVMOF-1. (**B**) Emission spectra of LVMOF-1 recorded upon sequential additions of gallic acid (10 μL of a 200 mg L^–1^ solution) to a 750 mg L^–1^ aqueous dispersion of LVMOF-1 (λ_exc_ = 306 nm)

The spectroscopic behaviour of LVMOF-1 was investigated to assess its suitability as a sensing material for antioxidant activity. As shown in Fig. 2A, the material exhibits an intense emission band centred at 615 nm upon excitation at 306 nm. While this luminescence profile appears consistent with that previously reported for LVMOF-1 by Gong et al. [23], a rigorous photophysical deconvolution reveals that this prominent peak (reaching *ca.* 140 in intensity) is actually a composite optical response. This feature is governed by a resonance convolution where the native, highly intense and hypersensitive ^5^D_0_ → ^7^F_2_ electric-dipole transition of Eu^3+^ (615 nm) directly overlaps with and cross-amplifies the grating-induced second-order scattering (SOS at *ca.* 612 nm) from the excitation monochromator.

To independently decouple these physical and electronic phenomena, diagnostic control experiments were performed by shifting the excitation wavelength to λ_exc_ = 290 nm (included as Fig. S1 in the ESM). As mathematically expected for a physical artifact, the SOS peak cleanly shifted to 580 nm (Fig. S1A), revealing its true, much lower physical scattering baseline (*ca.* 18) when the native Eu^3+^ emission at 615 nm drops to a basal level due to a lower energy-transfer efficiency under this configuration. Moreover, a magnified view of the baseline (Fig. S1B) reveals the independent, fixed emission bands of Eu^3+^ (^5^D_0_ → ^7^F_3_ at *ca.* 637 nm and ^5^D_0_ → ^7^F_4_ at *ca.* 680 nm), which remain consistently visible under both configurations owing to the stable magnetic-dipole character of these specific transitions. This eightfold intensity difference between the sintonized and decoupled configurations explicitly confirms that conventional ligand-sensitized antenna photoluminescence remains a primary driving force behind the overall analytical signal at 306 nm, which is instrumentally convoluted with the SOS band. Crucially, as displayed in Fig. 2B, the addition of increasing concentrations of gallic acid resulted in a pronounced, concentration-dependent quenching of this 615 nm composite peak. This attenuation profile may be rationalized by a simultaneous, multi-phenomenic mechanism driven by both chemical and physical processes. First, gallic acid possesses a strong ultraviolet absorption profile (260–280 nm) with a tail extending beyond 300 nm, which triggers a competitive IFE. This physical phenomenon attenuates the incident radiation, effectively reducing the photons available to both sensitize the Eu^3+^ metal centers (suppressing the dominant photoluminescence) and to be scattered by the colloidal crystalline framework. Second, this optical attenuation is likely assisted by specific host–guest interactions, which may include π-π stacking, charge transfer, and hydrogen bonding between the electron-rich phenolic groups of the analyte and the electron-deficient viologen-based linkers within the microporous channels of LVMOF-1. These interactions may induce a minor analyte-mediated micro-aggregation of the suspension. Given that Rayleigh scattering scales with the sixth power of particle diameter, this surface-mediated colloidal alteration directly modulates the physical SOS component of the convoluted peak.

Overall, these results demonstrate that the interaction between gallic acid and the LVMOF-1 framework produces a measurable and reproducible optical response primarily governed by a charge-transfer chemical quenching of the lanthanide photoluminescence, which may be concurrently reinforced by IFE and surface-mediated colloidal modulations of the SOS component. This multiphenomenic synergy highlights the potential of this material as a robust sensing platform for evaluating antioxidant activity.

### Analytical performance evaluation

The primary objective of this methodology is to serve as a total index method for evaluating the global total antioxidant capacity of complex food samples. In this context, GA is utilized strictly as a universal reference benchmark, allowing the total antioxidant activity of the samples to be quantitatively calibrated and expressed in terms of gallic acid equivalents (GAE). Notably, because the platform responds to the cumulative capacity of the matrix rather than to a single compound, the methodology can be easily generalized to other standard benchmarks (such as Trolox or ascorbic acid) to express the total antioxidant activity in their respective equivalents depending on the specific requirements of the assay. Hence, the analytical performance of the LVMOF-1-based spectroscopic assay was investigated. For this purpose, the relationship between the composite optical signal intensity and GA concentration was examined in order to characterize the sensing response of the MOF system. Key analytical parameters including linearity, sensitivity, precision, and detection limits were subsequently evaluated.

Prior to constructing the calibration model, the stabilization time of the analytical signal after each addition was assessed. The composite optical signal was monitored at different time intervals (10–190 s) for three representative gallic acid concentrations (0, 3.9, and 6.45 mg L^–1^). At 0 mg L^–1^, the signal showed a relative variation of approximately 6.8%. A similar behaviour was observed at 3.9 mg L^–1^, where the signal gives a variation of about 7.9%. In contrast, at 6.45 mg L^–1^ the analytical signal was considerably more stable, showing a variation below 1%. This increased stability at higher analyte concentrations can be attributed to the progressive occupation of interaction sites within the MOF framework, which leads to a more equilibrated host–guest system and reduces fluctuations in the analytical signal. These results indicate that the composite optical response is stable really fast and stable along the first 3 min. In any case, a waiting time of 120 s was selected before recording the fluorescence signal in all subsequent measurements to ensure adequate signal stabilization and improve measurement reproducibility.

The spectroscopic response of LVMOF-1 towards gallic acid was first evaluated using the Stern–Volmer model by monitoring the variation of the composite optical signal intensity ratio (*R* = *I*_*0*_*/I,* where *I*_*0*_ is the signal intensity of the blank MOF dispersion, and* I* is the signal intensity measured after addition of the analyte) as a function of the normalized gallic acid concentration (C^*^). In this work, the analyte concentration was expressed as the ratio between the gallic acid concentration (*C*_*GA*_) and the concentration of the MOF dispersion (*C*_*MOF*_) as defined in Eq. 3:

3 $${C}^{*}=\frac{{C}_{GA} (\mu g {L}^{-1})}{{C}_{MOF} (mg {L}^{-1})}$$

**Fig. 3:**
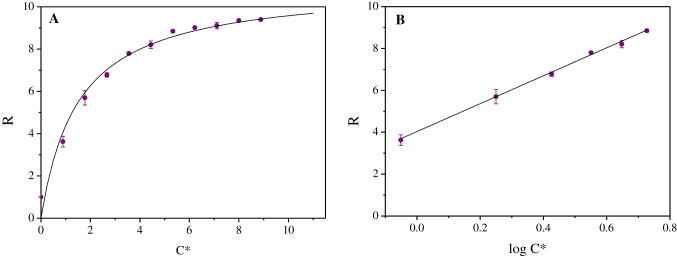
(A) Composite optical signal intensity ratio ratio of LVMOF-1 (*R*) as a function of normalized *C*_*GA*_ (C^*^) and fitting to a Langmuir-type model. (B) Calibration plot of *R* obtained against the logarithmic transformation of the normalized *C*_*GA*_ (C^*^). Error bars represent the standard deviation obtained from 3 independent calibration curves validated statistically across 3 different days (inter-day variability)

This normalization allows the signal to be related to the relative amount of analyte available per sensing material. As depicted in Fig. 3A, the composite optical signal intensity ratio (R) increases progressively with increasing analyte concentration, reflecting the quantitative interaction and optical attenuation processes between antioxidant molecules and the MOF framework. However, the experimental response gradually approaches a plateau at normalized concentrations above *ca.* 8 μg GA mg MOF^–1^ (~ 5.8 mg L^–1^ in *C*_*GA*_), indicating a saturation profile of the overall optical and host–guest interaction capacity. Such behaviour is typical of attenuation and host–guest systems involving porous materials and can be reasonably described by a Langmuir-type interaction model as shown in Eq. 4. This host–guest framework is preferred over conventional linear response models (such as the Stern–Volmer relationship) because it successfully accounts for the saturation of the available recognition sites within the framework architecture at higher analyte concentrations, reflecting a finite coordination or binding capacity.

4 $$R=\frac{{I}_{0}}{I}=\frac{{R}_{max}\cdot {C}^{*}}{{K}_{d}+ {C}^{*}}$$

The non-linear fitting of the experimental data to the Langmuir equation yielded a theoretical maximum signal ratio (*R*_*max*_) of *ca.* 11.03 and a half-saturation constant (*K*_*d*_) of *ca.* 1.52, with an excellent goodness of fit (R^2^ = 0.994). These results indicate that the overall optical sensing response is satisfactorily described by this model, reflecting a combined process where the chemical host–guest interactions within the finite capacity of the framework are structurally and mathematically convoluted with the concurrent physical attenuation phenomena (IFE and SOS modulations).

The saturation behaviour described by the Langmuir model limits the linear dynamic range (up to C_GA_ ~ 2 mg L^–1^) when the *R* is directly plotted against C^*^. To improve linearity and extend the usable calibration range, a logarithmic transformation of the normalized concentration was evaluated. The use of logarithmic transformation to linearize the response is commonly reported in interaction-based sensing systems where signal saturation occurs due to the finite thermodynamic or optical capacity of the platform [29]. In such systems, the logarithmic calibration compensates for the non-linear response caused by the convoluted contribution of adsorption, host–guest interactions, and physical attenuation mechanisms, allowing reliable quantification across a broader concentration range. When the *R* was plotted as a function of log (C^*^), a substantially improved linear relationship was obtained (Fig. 3B), following the equation:

5 $$R=6.51{\hspace{0.17em}}\mathrm{log} \left({C}^{*}\right)+4.03 ({R}^{2}=0.996)$$

Slight deviations from linearity were observed at the highest concentrations due to the progressive saturation of the overall optical sensing capacity, as predicted by the Langmuir model. Therefore, the upper boundary of the linear calibration interval was defined within the region where the regression still provided proportional response and minimal residual deviation. Under these conditions, the upper limit of the working range was established at C^*^ = 6.22 μg GA mg MOF^–1^, which corresponds to a *C*_*GA*_ of *ca.* 4.6 mg L^–1^. Defining this upper threshold is critically important for analytical users; because the logarithmic scale inherently compresses the signal response at high concentrations, any further expansion of the interval would lead to an unacceptable propagation of experimental error during concentration back-calculation, compromising the precision of the quantification.

The analytical performance of the LVMOF-1 spectroscopic assay was evaluated under the optimized conditions described in Sect. 2.8. The method exhibited excellent detection capability, providing a LOD of 0.0077 mg L^–1^ and a LOQ of 0.026 mg L^–1^, calculated from the standard deviation of the blank according to the IUPAC approach (3σ/S and 10σ/S, respectively). Reproducibility evaluated using standard solutions showed a relative standard deviation (RSD) below 7%, indicating the good precision of the optical measurements.

The analytical performance of the proposed LVMOF-1 assay was subsequently compared with several widely used antioxidant capacity methods, including the Folin–Ciocalteu, DPPH, ABTS and FRAP assays, whose experimental conditions are described in Sect. 2.5. As summarized in Table 2, the LVMOF-1 method provides substantially lower detection limits than most conventional colorimetric assays. In particular, the LOD obtained for LVMOF-1 is approximately two orders of magnitude lower than those of DPPH and FRAP, and markedly lower than that of the Folin–Ciocalteu assay. The sensitivity obtained in the linear region is also significantly higher than those of the classical methods, reflecting the strong composite optical response produced by the interaction between gallic acid and the MOF framework. Additionally, the proposed LVMOF-1 optical assay exhibited good precision, with RSD values ranging between 0.15 and 6.93%, which are comparable to those obtained with the FRAP assay (0.8–7.1%) and markedly lower than those observed for the Folin–Ciocalteu and DPPH methods. The ABTS assay showed slightly lower variability (0.1–1.8%), which may be attributed to the high stability of the ABTS•^+^ radical commonly reported for this method. Overall, these results highlight the strong analytical performance of the LVMOF-1 platform, particularly in terms of analytical sensitivity and detection capability.

**Table 2:** Comparison of analytical performance parameters for all evaluated antioxidant assays

ASSAY	LOD (mg L⁻^1^)	LOQ (mg L⁻^1^)	Sensitivity (L mg⁻^1^)	R^2^	RSD (%)
LVMOF-1	0.0077	0.026	6.51 ± 0.17 (log scale) 2.9 ± 0.3 (linear)	0.992	0.15–6.93
FOLIN–CIOCALTEU	0.358	1.193	0.052 ± 0.006	0.94	1–21
DPPH	1.451	4.836	0.053 ± 0.006	0.93	3.4–14.8
ABTS	0.015	0.049	0.092 ± 0.009	0.96	0.1–1.8
FRAP	1.420	4.734	0.051 ± 0.002	0.991	0.8–7.1

LOD and LOQ were calculated using the standard deviation of the blank (3σ_blank_ and 10σ_blank_, respectively). Sensitivity corresponds to the slope of the calibration curve. RSD = Relative Standard Deviation; R^2^ = coefficient of determination. For the LVMOF-1 assay, the linear sensitivity was calculated from the low-concentration linear region of the calibration curve (C_GA_ ≤ 2 mg L^–1^), whereas the log-scale sensitivity represents the constant slope of the linearized global model used for quantification across the entire extended range. All the other parameters for the LVMOF-1 assay were calculated from the log scale. All calibration curves were constructed using 6 concentration standards plus a blank. Measurements were performed across 3 independent calibration sequences prepared on 3 different days. The reported RSD ranges correspond to the minimum and maximum RSD values observed among the individual calibration points across the evaluated concentration intervals.

### Comparative evaluation of antioxidant assays using real samples

The proposed method to the analysis of real matrices operates as a total index method for evaluating global total antioxidant capacity, rather than as a selective tool for individual compound, such as GA. Consequently, the presence of structurally similar phenolic compounds in complex food samples does not constitute an analytical interference; rather, these molecules act as desired co-analytes that correctly contribute to the cumulative global signal, which is benchmarked against GA to yield reproducible GAE values. Furthermore, the platform demonstrated a remarkable capacity to minimize potential optical interferences from coloured compounds or natural food pigments. This high robustness against sample coloration represents a significant advantage over conventional UV–Vis colorimetric assays, which are heavily prone to spectral baseline shifts. In the proposed protocol, this is achieved because fluorescence detection inherently provides far greater selectivity and sensitivity against background absorption. Additionally, the measurement layout involves an exceptional sample dilution factor, where only 10 μL of the liquid sample (or its already diluted extract) are introduced into 3 mL of the sensing LVMOF-1 dispersion. This massive volume ratio dilutes any interfering matrix pigments to negligible concentration levels within the cuvette, preventing significant light attenuation. Nevertheless, to provide maximum analytical flexibility for exceptionally challenging or deeply coloured matrices, the method's single-vessel architecture can be easily adapted to a matrix-matched standard addition calibration (performing an additive calibration directly over the sample matrix), which seamlessly cancels out any residual matrix effect.

To evaluate the agreement between the proposed LVMOF-1 spectroscopic assay and conventional antioxidant capacity methods, a set of representative samples (see Sect. 2.2) was analysed using the five different assays (LVMOF-1, Folin–Ciocalteu, DPPH, ABTS and FRAP). The obtained antioxidant capacity values, expressed as gallic acid equivalents (GAE) are summarized in Table 3.

**Table 3:** Results of GAE in all samples, expressed as mean ± standard deviation

Sample	Antioxidant capacity (mg GAE kg^–1^)				
	*LVMOF-1*	Folin–Ciocalteu	DPPH	ABTS	FRAP
*Honeys (*mg GAE kg^–1^)					
H1	144 ± 10	1300 ± 30	119 ± 6	270 ± 10	2280 ± 120
H2	34 ± 2	340 ± 80	15.8 ± 0.3	13 ± 4	46 ± 6
H3	31.4 ± 1.3	197.2 ± 1.3	10.24 ± 0.16	5.2 ± 1.3	38 ± 2
*Tea (*mg GAE g^–1^)					
T1	11.6 ± 0.2	53.52 ± 0.08	9.76 ± 0.13	48 ± 4	34 ± 2
T2	2.92 ± 0.12	26.7 ± 0.7	4.09 ± 0.06	12.39 ± 0.10	16.4 ± 1.1
T3	1.61 ± 0.09	21.1 ± 0.6	3.2 ± 0.7	9.02 ± 0.04	11.5 ± 0.8
*Wines (*mg GAE kg^–1^)					
W1	955 ± 12	3654.3 ± 1.0	270 ± 11	617.8 ± 1.5	1490 ± 99
W2	35.1 ± 0.4	201 ± 5	15.2 ± 0.3	74.9 ± 0.4	52 ± 11
W3	86.9 ± 1.1	225.5 ± 1.5	38 ± 2	101.4 ± 1.6	188 ± 14

Note: Units for antioxidant capacity of the tea are expressed as mg GAE g^–1^ since they are calculated over solid dry leaf extracts, while honey and wines are calculated over the liquid samples

As expected, the absolute antioxidant capacity values varied considerably depending on the analytical method used, reflecting the different chemical principles underlying each assay. In general, the Folin–Ciocalteu and FRAP methods provided the highest values, whereas the DPPH and ABTS assays yielded comparatively lower values. The LVMOF-1 fluorescence assay produced intermediate values while preserving similar trends across the analysed samples. Consequently, absolute GAE values are highly method-dependent and not directly interchangeable between different analytical platforms, as each assay probes distinct chemical pathways and features specific reaction kinetics. To further evaluate the reproducibility of the different assays in real matrices, the RSD associated with each measurement was calculated from the replicate determinations reported in Table 3. The LVMOF-1 spectroscopic assay exhibited consistently low variability across the analysed samples, with RSD values ranging from *ca.* 1.3 to 6.0%. In contrast, the conventional colorimetric assays showed considerably wider RSD ranges, reaching *ca.* 1.2–24% for Folin–Ciocalteu, *ca.* 1.6–22% for DPPH, *ca.* 0.6–31% for ABTS, and *ca.* 4–19% for FRAP. These results indicate that the LVMOF-1 method provides more stable and reproducible responses in complex sample matrices, highlighting the robustness of the optical-based detection approach compared with conventional radical or redox assays.

To evaluate whether the different antioxidant assays provided consistent rankings of the analysed samples, a non-parametric Friedman test followed by Kendall’s coefficient of concordance (W) was applied. The Friedman test assesses whether statistically significant differences exist among several related measurement methods when applied to the same set of samples, while Kendall’s W quantifies the degree of agreement among the rankings generated by those methods. The Friedman test revealed significant differences between the analytical assays (χ^2^ = 27.64, df = 4, p < 0.001), confirming that the absolute antioxidant capacity values depend on the chemical principles underlying each method. However, Kendall’s coefficient of concordance indicated a moderate level of agreement among the assays (W = 0.415), demonstrating that although the methods provide a rough trend in sample ranking, this moderate value highlights that distinct methodological features and reaction mechanisms prevent a fully consistent classification across the different platforms. To further assess the contribution of each method to the overall concordance, a sensitivity analysis was performed by recalculating W after sequentially excluding each assay. The removal of any individual method led to a decrease in concordance, indicating that all assays contribute positively to the overall ranking consistency. In particular, the exclusion of the Folin–Ciocalteu method resulted in the most pronounced reduction (W = 0.247), highlighting its key role in preserving the global ranking of antioxidant capacity. Similarly, the removal of the FRAP method led to a notable decrease in W (0.297), suggesting that it also contributes significantly to ranking agreement despite its distinct response behaviour observed in other analyses. The exclusion of the ABTS method produced a moderate decrease in concordance (W = 0.376), while the LVMOF-1 spectroscopic assay showed a comparable level of agreement with the rest of the methods, as its removal resulted in only a slight reduction in W (0.334). Overall, these results confirm that the LVMOF-1 assay captures antioxidant trends broadly comparable to those obtained using conventional methods, although the overall ranking agreement among assays is moderate rather than strong, reflecting the influence of their different underlying chemical mechanisms.

**Fig. 4:**
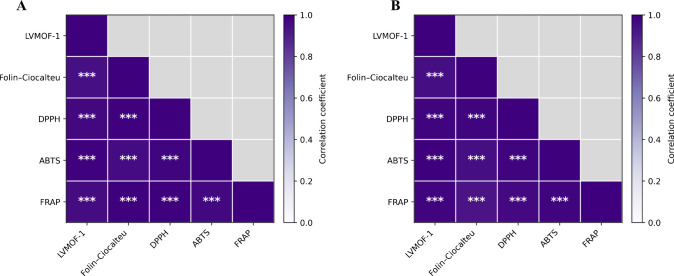
Pearson (**A**) and Spearman (**B**) correlation heatmaps describing the relationships among the antioxidant assays evaluated in this work

The relationships between the different antioxidant assays were examined using Pearson and Spearman correlation analyses, and the results are summarized in the heatmaps shown in Fig. 4A and 4B.

The Pearson correlation heatmap (Fig. 4A) revealed very strong linear relationships between the LVMOF-1 spectroscopic assay and the conventional antioxidant methods, with correlation coefficients ranging from 0.947 to 0.996 (p < 0.001 in all cases). In particular, very strong correlations were observed with the ABTS (r = 0.996) and DPPH (r = 0.969) assays, followed by FRAP (r = 0.959) and Folin–Ciocalteu (r = 0.947). These results indicate that the proposed optical-based approach closely follows the trends in antioxidant capacity obtained using widely established methodologies, although it is paramount to emphasize that such high mathematical correlation does not imply perfect analytical equivalence or quantitative interchangeability between the methods. A similar pattern was observed in the Spearman correlation heatmap (Fig. 4B), where all methods exhibited very strong monotonic relationships (ρ = 0.933–1.000, p < 0.001), confirming that the ranking of samples is highly consistent across the evaluated techniques. Given the wide concentration range among sample groups, particularly due to the high values observed for T samples, additional analyses were performed to assess the potential influence of scale effects on the observed correlations. When the T samples were excluded, the correlations remained very strong (r = 0.94–0.99), confirming that the observed relationships are not solely driven by extreme values but reflect consistent trends between methods.

Strong correlations were also observed among the conventional assays themselves (r ≥ 0.961), indicating consistent behaviour across methods despite their different underlying chemical principles. The Folin–Ciocalteu and FRAP methods are based on electron transfer reactions, while ABTS also predominantly follows electron transfer mechanisms, with possible contributions from hydrogen atom transfer. In contrast, the DPPH assay is mainly governed by radical scavenging processes involving hydrogen atom transfer. While these strong mathematical correlations demonstrate that the LVMOF-1 composite optical response successfully captures the same global antioxidant trends as the traditional assays, they do not imply that the underlying molecular sensing mechanism operates via identical redox pathways. Instead, the platform responds empirically to the cumulative presence of these reducing co-analytes through its specific host–guest and optical attenuation framework.

Although correlation analyses provide valuable information about the relationships between analytical methods, they do not necessarily indicate whether two techniques yield comparable quantitative results. Therefore, a Bland–Altman analysis was performed to evaluate the agreement between the LVMOF-1 spectroscopic assay and the conventional antioxidant capacity methods. This approach assesses the systematic bias between two methods and determines the range within which the majority of the differences between measurements are expected to lie.

For each pair of methods, the mean value between the two measurements and their difference were calculated as:

6 $$Average= \frac{{GAE}_{LVMOF-1}+{GAE}_{classic method}}{2}$$

7 $$Difference= {GAE}_{LVMOF-1}-{GAE}_{classic method}$$

where ${GAE}_{LVMOF-1}$ corresponds to the antioxidant capacity values obtained by the LVMOF-1 assay and the ${GAE}_{classic method}$ to the antioxidant capacity values obtained by the classic method that is being compared. The average difference between both methods (Bias) was then calculated as:

8 $$Bias= \overline{Difference }$$

The limits of agreement were defined as:

9 $$Upper Limit=Bias+1.96\cdot {s}_{differences}$$

10 $$Lower Limit=Bias-1.96\cdot {s}_{differences}$$

where ${s}_{differences}$ represents the standard deviation of the differences. These limits indicate the interval within which approximately 95% of the differences between methods are expected to fall.

**Fig. 5:**
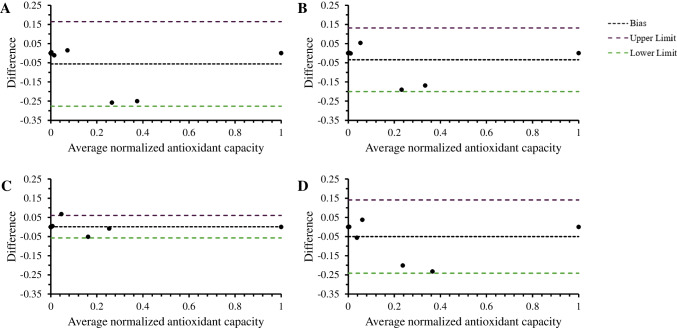
Bland–Altman plots comparing the antioxidant capacity values obtained with the LVMOF-1 fluorescence assay and the conventional methods: (A) Folin–Ciocalteu, (B) DPPH, (C) ABTS and (D) FRAP. The dashed lines represent the mean difference (bias) and the limits of agreement (Bias ± 1.96 ${s}_{differences}$)

The Bland–Altman plots obtained for the comparisons between the LVMOF-1 assay and the conventional methods are presented in Fig. 5. Despite the strong correlations observed for all methods, this analysis allows the evaluation of their quantitative agreement. For the Folin–Ciocalteu method (Fig. 5A), a moderate negative bias was observed (*ca.* − 0.056, normalized units), with relatively wide limits of agreement (*ca.* − 0.277 to 0.165), indicating noticeable dispersion between both methods. A similar behaviour was observed for the FRAP assay (Fig. 5D), which also showed a negative bias (*ca.* − 0.050) and comparably wide limits of agreement (*ca.* − 0.241 to 0.141), suggesting increased variability in the quantitative agreement. In contrast, the comparison with the DPPH method (Fig. 5B) exhibited a smaller bias (*ca.* − 0.034) and narrower limits of agreement (*ca.* − 0.200 to 0.132), indicating improved agreement between both methods. Notably, the ABTS assay (Fig. 5C) showed the best agreement with the LVMOF-1 spectroscopic method, with a negligible bias (*ca.* 0.001) and markedly narrower limits of agreement (*ca.* − 0.057 to 0.060), reflecting a high level of quantitative consistency. These results indicate that, despite the strong correlations observed between all methods, differences in absolute response remain method-dependent. In particular, the narrower limits of agreement observed for ABTS suggest a closer alignment with the LVMOF-1 optical response, whereas Folin–Ciocalteu and FRAP exhibit greater variability. Overall, the relatively small bias values confirm the absence of significant systematic deviations, while the differences in limits of agreement highlight the influence of method-specific response characteristics.

Principal component analysis (PCA) was applied to obtain an integrated overview of the relationships among the different antioxidant assays and to identify potential patterns in their responses. Prior to performing PCA, the suitability of the dataset was evaluated using the Kaiser–Meyer–Olkin (KMO) measure of sampling adequacy and Bartlett’s test of sphericity. The obtained KMO value (0.621) indicated an acceptable sampling adequacy, while Bartlett’s test was highly significant (χ^2^ = 126.05, p < 0.001), confirming that the correlation matrix was appropriate for multivariate analysis.

**Fig. 6:**
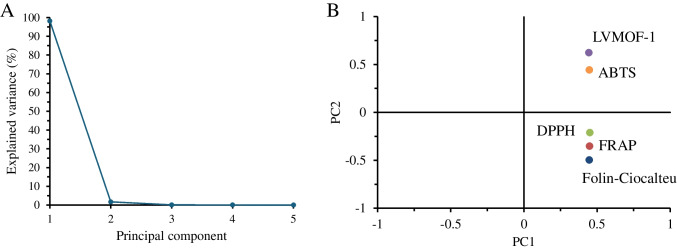
Principal component analysis (PCA) of the antioxidant assays. (A) Scree plot showing the explained varience associated with each principal component. (B) Distribution of the antioxidant methods in the PC1–PC2 space according to their loading values

The PCA results (Fig. 6A) revealed that the first principal component (PC1) accounts for the vast majority of the total variance (98.2%), with an eigenvalue of 5.52, indicating that the overall antioxidant capacity can be described by a single dominant factor. In contrast, the second component (PC2) explains only a marginal proportion of the variance (1.69%), suggesting that differences among methods are relatively minor. The loading plot (Fig. 6B) shows that all antioxidant assays exhibit very similar and high contributions to PC1, confirming a highly consistent global response across methods. The LVMOF-1 spectroscopic assay is closely aligned with the cluster defined by the conventional assays, indicating that it follows the same overall antioxidant trend.

Although PC2 contributes only a small fraction of the variance, it provides additional insight into subtle differences between methods. In this dimension, LVMOF-1 (loading = 0.623 on PC2) and ABTS (loading = 0.443 on PC2) appear located on the same side of the component, whereas Folin–Ciocalteu (− 0.496 on PC2), DPPH (− 0.211 on PC2) and FRAP (− 0.352 on PC2) are positioned in the opposite direction. This slight separation suggests a closer similarity between the LVMOF-1 optical response and the ABTS assay, although the effect remains minor due to the limited variance explained by PC2. Notably, this observation is consistent with the Bland–Altman analysis, where ABTS exhibited the smallest bias and the narrowest limits of agreement with respect to LVMOF-1, indicating the highest level of quantitative agreement among the evaluated methods.

Overall, these results confirm that the LVMOF-1 spectroscopic assay captures the same underlying antioxidant capacity dimension as conventional methods, as reflected by the dominant contribution of PC1, while also revealing subtle method-dependent differences. The combined evidence from correlation analysis, Bland–Altman comparison and PCA indicates that the MOF-based optical platform provides results that are strongly correlated, and highly consistent in terms of sample ranking and trend tracking, although the inherent method-dependent differences restrict direct quantitative comparisons at the absolute concentration level.

### Greenness evaluation of the antioxidant methods

**Fig. 7:**
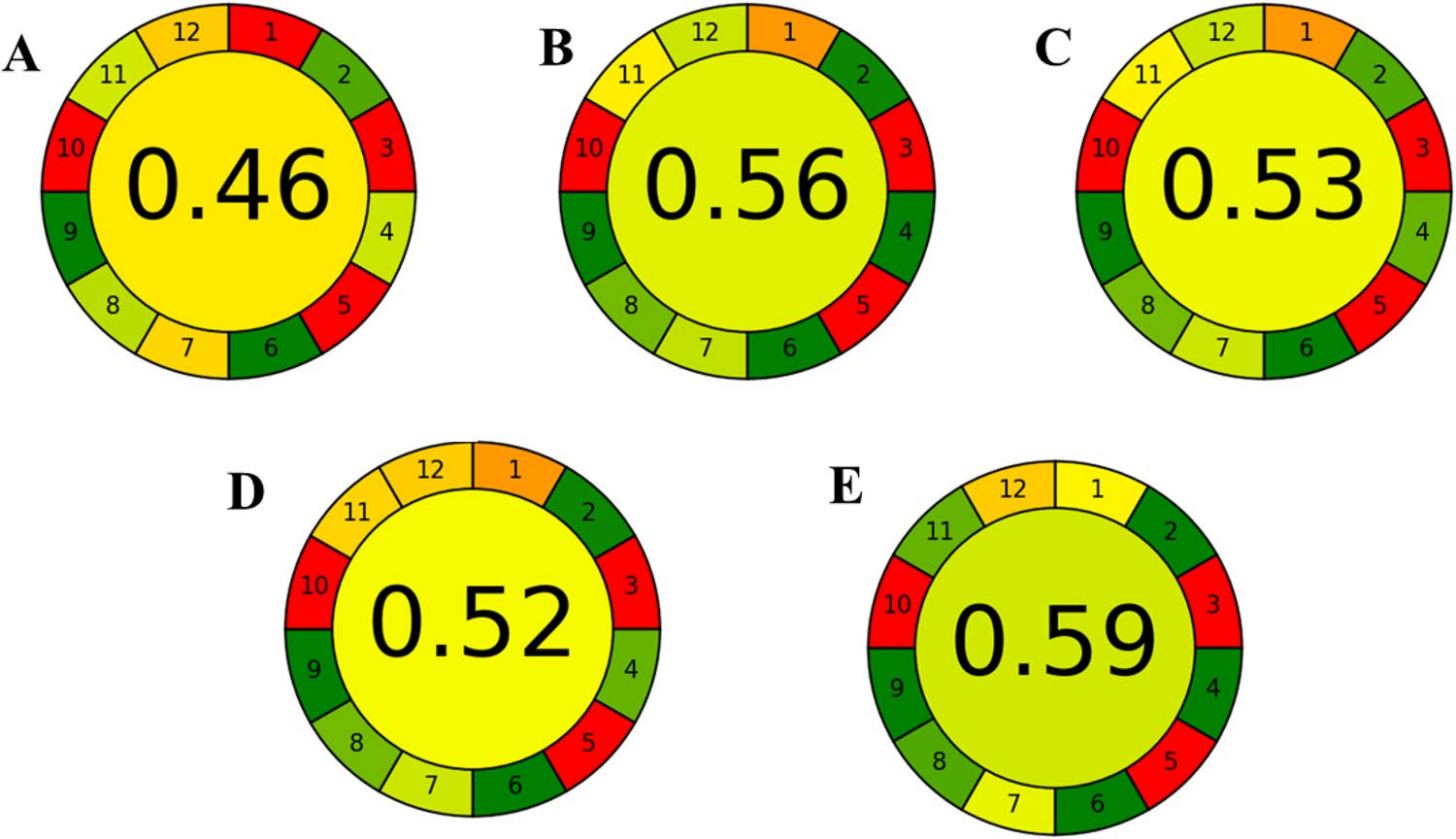
AGREE pictograms and overall greenness scores for the five antioxidant methods evaluated: (**a**) Folin–Ciocalteu, (**b**) DPPH, (**c**) ABTS, (**d**) FRAP, and (**e**) LVMOF-1 fluorescence assay. The AGREE reports for each method are provided in the ESM

To comprehensively evaluate the environmental sustainability and alignment with Green Analytical Chemistry (GAC) principles of the different antioxidant capacity assays (Folin–Ciocalteu, DPPH, ABTS, FRAP, and the proposed LVMOF-1 method), the AGREE (Analytical GREEnness) metric was employed (Fig. 7, the AGREE reports for each method are provided in the ESM). This approach, which converts the twelve principles of GAC into a final consensus score and a visual 0–1 scale, was applied using the user-friendly, free software tool developed by Pena-Pereira, et al. [30]. The evaluation considered key analytical parameters for each method, including sample and reagent volumes, consumption of toxic solvents, waste generation, analysis time, energy requirements, and operator's safety, transforming them into a quantitative greenness profile for direct comparison.

The LVMOF-1 spectroscopic assay achieved the highest AGREE score (0.59), reflecting its strong advantages from a green chemistry perspective (Fig. 7). Notably, it completely avoids the use of toxic organic solvents such as methanol, required in large quantities in DPPH and ABTS assays (up to 1.45 mL per analysis). It also requires a much smaller sample volume (10 μL), reducing overall reagent consumption. Additionally, its short stabilization time (2 min) eliminates long incubation steps (60 min in Folin–Ciocalteu and DPPH) and avoids heating requirements such as those in FRAP (37 °C), resulting in lower energy use and minimal waste generation. Furthermore, unlike traditional colorimetric methods that require separate cuvettes or wells for each calibration or sample point, the LVMOF-1 method allows sequential measurements in the same vessel, significantly reducing consumable waste and sample handling. Altogether, these features position the LVMOF-1 optical assay as a more sustainable and operationally efficient alternative for evaluating antioxidant capacity.

### Economic assessment

A comparative economic evaluation of the five antioxidant capacity assays was performed to assess their cost-effectiveness and operational feasibility (Table 4). The analysis considered three main components: (i) reagent consumption, (ii) energy requirements, and (iii) active personnel time.

**Table 4:** Cost breakdown per sample (in €) for the five evaluated antioxidant assays

Assay	Assay cost (€/sample)			
	Reagents	Energy	Personnel	Total
Folin–Ciocalteu	0.7044	0.0001	2.1140	2.8185
DPPH	0.0282	0.0001	0.7047	0.7330
ABTS	0.5871	0.0001	2.1140	2.7012
FRAP	0.2384	0.0034	2.1140	2.3558
LVMOF-1	0.2801	0.0001	0.3523	0.6325

Reagent costs were calculated from current commercial prices (obtained from major suppliers, see Table S1 in the Supporting Information). For the LVMOF-1 assay, the cost of the sensing material was derived from the complete synthesis process, including the preparation of the viologen-based ligand, the solvothermal synthesis of the MOF, and the active labour time involved in these steps, all amortised over the final mass of LVMOF-1 obtained (see Table S1, Supporting Information). This material cost was then used to calculate the cost of the aqueous dispersion used in the measurements. Energy costs were derived from the power ratings of the instruments used (UV–Vis spectrophotometer: 0.1 kW; spectrofluorimeter: 0.15 kW; water bath: 0.3 kW), the duration of each measurement or incubation step, and the local electricity tariff (0.0655 € kWh^–1^). Personnel costs were calculated using the average salary of a laboratory technician in Spain (21.14 € h^–1^), considering only the active manipulation time (sample preparation and instrument reading), since incubation periods do not require constant operator attention. During the total protocol time, multiple samples can be processed in parallel. Therefore, the number of samples handled per protocol cycle was determined from the ratio of the total protocol time to the active time per sample. The personnel cost per sample was obtained by multiplying the hourly labour cost by the total protocol time and dividing by the number of samples processed within that period. Total protocol and active times were taken from the procedures in Sects. 2.5 and 2.8 and summarised in Table S2.

The LVMOF-1 spectroscopic assay exhibited the lowest total cost per sample (0.63 €), followed by DPPH (0.73 €), FRAP (2.36 €), ABTS (2.70 €), and Folin–Ciocalteu (2.82 €). The higher cost of the Folin–Ciocalteu method is mainly attributable to its longer active handling time (6 min per sample) and greater reagent consumption. In contrast, FRAP and ABTS showed intermediate personnel costs due to their comparable active manipulation times (around 6 min). Although the reagent cost for LVMOF-1 (0.280 €) was slightly higher than that of DPPH owing to the multistep synthesis of the MOF, the overall cost remained the lowest because of its very short active handling time (1 min). Moreover, the LVMOF-1 assay enables sequential measurements in the same cuvette, further reducing consumable waste when multiple samples are analysed. Overall, the economic assessment confirms that the proposed LVMOF-1 method is not only the most cost-effective among the evaluated assays but also aligns with the greenness advantages highlighted in the AGREE evaluation (Sect. 3.5), making it a sustainable and practical alternative for routine antioxidant screening in food samples.

## Conclusions

This work demonstrates that functional luminescent MOFs can be effectively translated into practical analytical tools optical sensing platform for antioxidant capacity assessment. The LVMOF-1 platform provides a sensitive and reproducible composite optical response (molecular quenching, IFE, and colloidal SOS) towards phenolic antioxidants, with detection limits in the low µg L^–1^ range and variability consistently below 7%, supporting its suitability for quantitative applications.

From an analytical perspective, the method shows strong consistency with established assays, as demonstrated by the moderate concordance obtained (Kendall’s W = 0.415) and the strong correlations with conventional methods (Pearson r = 0.947–0.996). Agreement analysis further confirmed the comparability of results, with low bias and relatively narrow limits of agreement in Bland–Altman plots for most methods. In addition, PCA revealed that the LVMOF-1 spectroscopic response closely follows the dominant variance pattern defined by classical assays, confirming that it captures the same underlying antioxidant trends despite differences in absolute values. At the same time, the method offers clear operational advantages, including significantly shorter analysis times (≤ 2 min) and improved robustness in complex matrices, reducing susceptibility to matrix interferences.

Importantly, the proposed approach stands out in terms of practical implementation. The low cost per analysis (0.63 € per sample) and favourable greenness profile highlight its potential for routine use in quality control laboratories, where rapid, cost-effective, and reproducible measurements are essential. In this context, further reduction of costs could be envisaged through the commercialization of the MOF material, facilitating its direct use without the need for in-house synthesis.

Beyond its immediate application, this work opens new avenues for integrating functional materials such as MOFs into routine analytical methodologies. The combination of tuneable host–guest interactions, rapid response, and simplified workflows provide a promising framework for the development of next-generation alternatives to classical antioxidant assays.

## Supplementary Information

Below is the link to the electronic supplementary material.

Supplementary file1 (PDF 298 KB)

## Data Availability

Data will be made available on request.
