# Supplementary material for: Lanthanide-based luminescent metal–organic framework as an optical sensing platform for rapid and reliable assessment of antioxidant activity in food samples
Source: Mikrochim Acta. 2026 Jul 21;193(8):556. doi: 10.1007/s00604-026-08244-8 (PMC13384980; doi:10.1007/s00604-026-08244-8)
Supplement: Supplementary file 1 — Supplementary file1 (PDF 298 KB) [file 604_2026_8244_MOESM1_ESM.pdf]

Lanthanide-based luminescent metal–organic framework as an optical sensing platform for rapid and reliable assessment of antioxidant activity in food samples

Neus Crespí-Sánchez[1], Francesc Amaro Simeon-Antich[2], Ernesto Francisco Simó-Alfonso[2], Enrique Javier Carrasco-Correa[2,*]

[1] Department of Chemistry, University of the Balearic Islands, Cra. de Valldemossa, km 7.5, 07122-Palma de Mallorca, Spain

[2] CLECEM group, Department of Analytical Chemistry, Faculty of Chemistry, University of València, Avenida Vicent Andrés Estellés, 19, 46100-Burjassot, València, Spain

*Corresponding author:

Dr. Enrique Javier Carrasco-Correa

e-mail: enrique.carrasco@uv.es

Tel.: +34963544248

Fax: +34963544436

Pages: 6

Figures: 1

Tables: 2

Conventional antioxidant capacity assays

Folin-Ciocalteu assay

The assay was adapted from the method described by Oliviera et al. [1]. A mixture of 250 µL of gallic acid standard or sample solution, 2 mL of ultrapure water, and 500 µL of commercial Folin-Ciocalteu reagent was prepared. After centrifugation and 3 min of resting, 5 mL of 20% (m/v) sodium carbonate solution were added, adjusting the pH to ~10. The mixture was kept at room temperature in the dark for 1 h before measuring absorbance at 765 nm.

DPPH assay

Following the method described by Oliviera et al. [1], 100 µL of standard or sample solution, 100 µL of MeOH, and 1.5 mL of 40 mg L^{-1} DPPH in MeOH:H_2O (8:2, v/v) were mixed. The reaction mixture was kept in the dark at room temperature for 1 h and absorbance was measured at 517 nm.

ABTS assay

Adapted from the methods of Anand et al. and Fernández-Segovia et al. [2, 3], 250 µL of standard or sample solution, 250 µL of MeOH, and 1.5 mL of ABTS solution were mixed. The mixture was incubated at room temperature in the dark for 20 min, and the absorbance was measured at 734 nm. The ABTS reagent was prepared 24 h before use by mixing 7 mM ABTS and 2.45 mM $K_2S_2O_8$ in ultrapure water, stored at room temperature in the dark, and diluted in MeOH up to get an absorbance of 0.700 ± 0.020 at 734 nm.

FRAP assay

Following Anand et al. [3], 100 µL of standard or sample solution, 100 µL of MeOH, and 1.8 mL of FRAP reagent were mixed and incubated at 37 °C in a water bath for 10 min. Absorbance was measured at 593 nm. The FRAP reagent was prepared by mixing 3 M

acetate buffer (pH 3.6), 10 mM TPTZ in 40 mM HCl, and 20 mM $FeCl_3\cdot 6H_2O$ in a 10:1:1 ratio.

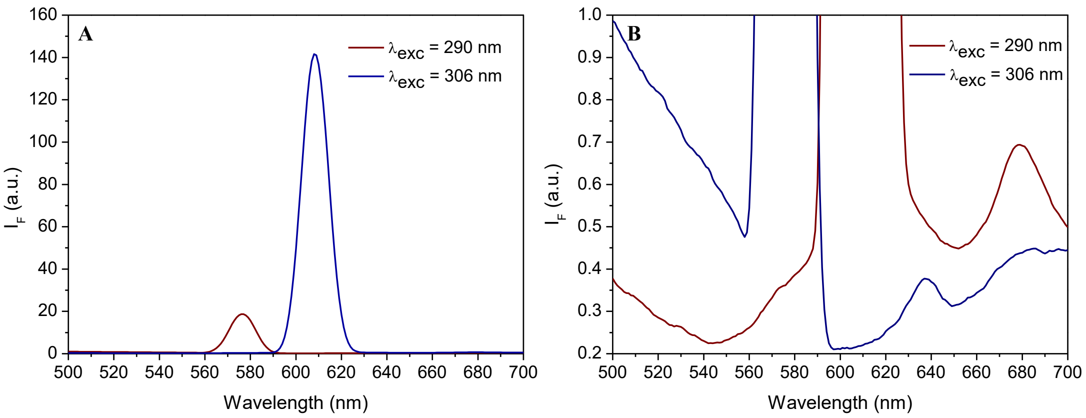

Fig. S1. Fluorescence emission spectra of the sensing platform recorded at two different excitation wavelengths to differentiate true photoluminescence from scattering artifacts: (A) Full-scale spectra showing the prominent second-order scattering (SOS) peaks at 580 and 612 nm corresponding to the λ_{exc} = 290 and 306 nm optical profile, respectively. (B) Magnified view of the spectral baseline highlighting the corresponding emission bands of LVMOF-1.

Table S1. Economic Basis for the Preparation of LVMOF-1 Dispersions

	Ligand synthesis		
**Reagents**	**Quantity (mL or g)**	**Unit cost (€/L or €/g)**	**Cost (€)**
4,4′-Bipyridine	1 g	0.0301 €/g	0.0301
Dimethyl 5-(bromomethyl)isophthalate solution	3.67 g	0.8380 €/g	3.0755
Acetonitrile	17 mL	15.0000 €/L	0.2550
HCl 37%	30 mL	11.1360 €/L	0.3341

Ligand obtained (g)	Total cost (€)	Cost (€/g)
1.5	3.6946	2.4631

	MOF synthesis		
**Reagents**	**Quantity (mL or g)**	**Unit cost (€/L or €/g)**	**Cost (€)**
Ligand	0.05 g	2.4631 €/g	0.1232
$EuCl_3 \cdot 6H_2O$ solution	0.049 g	2.1140 €/g	0.1036
Acetonitrile	2 mL	15.0000 €/L	0.0300
Water	1 mL	0.0000 €/L	0.0000

LVMOF-1 obtained (g)	Reagent cost (€)	Personnel cost (€)	Total cost (€)	Cost (€/mg)
0.07	0.2567	8.4560	8.7127	0.1245

MOF concentration (mg/mL)	Dispersion cost (€/mL)
0.7500	0.0934

Table S2. Detailed Supplementary Cost Analysis of the Evaluated Antioxidant Assays

Folin–Ciocalteu					
**Reagents**	**Quantity (mL)**	**Unit cost (€/mL)**	**Cost (€/sample)**		
Folin reagent	0.5	0.2334	0.1167		
20% Na_2CO_3 solution	5	0.1175	0.5877		
Ultrapure water	2	0.0000	0.0000		
**Energy**	**Quantity (min)**	**Unit cost (€/min)**	**Cost (€/sample)**		
UV-Vis measurement	1	0.0001	0.0001		
**Personnel**	**Quantity (min)**	**Unit cost (€/min)**	**Active time (min)**	**Number of Sample**	**Cost (€/sample)**
Protocol time	70	0.3523	6	11	2.1140

DPPH					
**Reagents**	**Quantity (mL)**	**Unit cost (€/mL)**	**Cost (€/sample)**		
Methanol	0.1	0.0443	0.0044		
DPPH working solution	1.5	0.0159	0.0238		
**Energy**	**Quantity (min)**	**Unit cost (€/min)**	**Cost (€/sample)**		
UV-Vis measurement	1	0.0001	0.0001		
**Personnel**	**Quantity (min)**	**Unit cost (€/min)**	**Active time (min)**	**Number of samples**	**Cost (€/sample)**
Protocol time	62	0.3523	2	31	0.7047

ABTS					
**Reagents**	**Quantity (mL)**	**Unit cost (€/mL)**	**Cost (€/sample)**		
Methanol	0.25	0.0443	0.0111		
ABTS working solution	1.5	0.3840	0.5760		
**Energy**	**Quantity (min)**	**Unit cost (€/min)**	**Cost (€/sample)**		
UV-Vis measurement	1	0.0001	0.0001		
**Personnel**	**Quantity (min)**	**Unit cost (€/min)**	**Active time (min)**	**Number of Sample**	**Cost (€/sample)**
Protocol time	22	0.3523	6	3	2.1140

FRAP					
**Reagents**	**Quantity (mL)**	**Unit cost (€/mL)**	**Cost (€/sample)**		
Methanol	0.1	0.0443	0.0044		
FRAP Reagent	1.8	0.1300	0.2340		
**Energy**	**Quantity (min)**	**Unit cost (€/min)**	**Cost (€/sample)**		
Water bath	10	0.0003	0.0033		
UV-Vis measurement	1	0.0001	0.0001		
**Personnel**	**Quantity (min)**	**Unit cost (€/min)**	**Active time (min)**	**Number of Sample**	**Cost (€/sample)**
Protocol time	12	0.3523	6	2	2.1140

	LVMOF-1				
**Reagents**	**Quantity (mL)**	**Unit cost (€/mL)**	**Cost (€/sample)**		
LVMOF-1 dispersion	3	0.0934	0.2801		
**Energy**	**Quantity (min)**	**Unit cost (€/min)**	**Cost (€/sample)**		
Fluorescence measurement	0.5	0.0002	0.0001		
**Personnel**	**Quantity (min)**	**Unit cost (€/min)**	**Active time (min)**	**Number of Sample**	**Cost (€/sample)**
Protocol time	5	0.3523	1	5	0.6325

References

1. De Oliveira Neto JR, Rezende SG, Lobón GS, et al (2017) Electroanalysis and laccase-based biosensor on the determination of phenolic content and antioxidant power of honey samples. Food Chemistry 237:1118–1123. https://doi.org/10.1016/j.foodchem.2017.06.010

2. Anand S, Pang E, Livanos G, Mantri N (2018) Characterization of Physico-Chemical Properties and Antioxidant Capacities of Bioactive Honey Produced from Australian Grown Agastache rugosa and its Correlation with Colour and Poly-Phenol Content. Molecules 23:108. https://doi.org/10.3390/molecules23010108

3. Fernández-Segovia I, Lerma-García MJ, Fuentes A, Barat JM (2018) Characterization of Spanish powdered seaweeds: Composition, antioxidant capacity and technological properties. Food Research International 111:212–219. https://doi.org/10.1016/j.foodres.2018.05.037
